# Progression of coronary atherosclerotic plaque burden and relationship with adverse cardiovascular event in asymptomatic diabetic patients

**DOI:** 10.1186/s12872-019-1016-4

**Published:** 2019-02-11

**Authors:** Junjie Yang, Guanhua Dou, Christian Tesche, Carlo N. De Cecco, Brian E. Jacobs, U. Joseph Schoepf, Yundai Chen

**Affiliations:** 1 Department of Cardiology, Chinese PLA General Hospital, Beijing, People’s Republic of China; 2 Master Program of Medical Science and Clinical Investigation, Harvard Medical School, Boston, MA USA; 3 Division of Cardiovascular Imaging, Department of Radiology and Radiological Science, Medical University of South Carolina, Charleston, SC USA; 4 Department of Cardiology and Intensive Care Medicine, Heart Center Munich- Bogenhausen, Munich, Germany

**Keywords:** Coronary artery disease, Type 2 diabetes, Coronary computed tomography angiography, Plaque progression, Outcome

## Abstract

**Background:**

The heterogeneity of risk in patients with diabetes mellitus (DM) is acknowledged in new guidelines promulgating different treatment recommendations for diabetics at low cardiac risk. We performed a retrospective longitudinal follow-up study to evaluate coronary plaque progression and its impact on cardiac events in asymptomatic diabetic patients.

**Methods:**

Data of 197 asymptomatic patients (63.1 ± 17 years, 60% males) with DM and suspected coronary artery disease (CAD) who underwent clinically indicated dual-source cardiac computed tomography (CT) were retrospectively analyzed. Patients with DM received standard of care treatment. Patients were classified into two groups based on CT coronary artery calcium scores (CACS): A, CACS> 10; B, CACS≤10. Progression of coronary plaque burden in both groups was evaluated and compared by baseline and follow-up coronary CT angiography (CCTA) using semi-automated plaque analysis and quantification software. Follow-up data were retrospectively gathered from medical records and endpoints of cardiac events were recorded via prospective phone-calls. The impacts of plaque composition and progression on cardiac events were specifically assessed.

**Results:**

Patients with CACS> 10 showed an increase in dense coronary calcium volume, while patients with CACS≤10 had a more pronounced increase in the volume of low-attenuation “lipid-rich” plaque components between CCTA acquisitions. The composite endpoint occurred in 20 patients (10.2%) after a median follow-up period of 41.8 months. Furthermore, at follow-up CCTA, the presence of CACS> 10 (adjusted odds ratio, 0.701; 95% CI, 0.612–0.836), increase of dense calcium volume (OR, 0.860 95% CI, 0.771–0.960), and lipid volume (OR, 1.013; 95% CI, 1.007–1.020) were all independent predictors of cardiac events.

**Conclusion:**

Asymptomatic patients with DM experienced plaque progression as well as progression to “overt or silent CAD”. The relative increase in plaque volume was associated with subsequent cardiac events, and the coronary calcification seemed to be inversely related to the outcome in asymptomatic diabetic patients.

## Highlights

- Serial non-invasive CCTA enables quantification of atherosclerotic plaque progression in asymptomatic diabetic patients.
- Changes in low attenuation atheroma volume as an imaging biomarker of vulnerable plaque may help to identify patients at risk.
- Higher levels of atherosclerotic calcification may have protective value on plaque stabilization.

## Background

Diabetes mellitus (DM) has been widely considered a coronary artery disease (CAD) equivalent, which implies a 10-year cardiovascular risk of 20% for every diabetic patient [1]; however, recently published data refute this notion [2, 3]. According to medical convention, patients with DM are at a higher risk for developing CAD, despite lacking any pertinent symptoms [4]. Thus, it remains controversial whether asymptomatic diabetic patients should be screened for CAD and receive early optimized medical therapy even there is low coronary calcium score [5, 6].Previous studies have demonstrated that diabetic patients with initially asymptomatic CAD show accelerated plaque progression, despite the use of optimized medical treatment. Eventually, patients with DM develop more major adverse cardiac events (MACE) and evidence of permanent myocardial injury than patients without [7, 8]. Recent studies have highlighted the incremental increase in event rates associated with coronary artery calcification [9]. These investigations showed significantly more adverse outcomes in diabetic patients with a computed tomographic (CT) coronary artery calcium score (CACS) ≥10 than in patients without DM [10]. Thus, effective screening strategies for appropriate cardiovascular risk stratification of asymptomatic diabetic patients warrant further study.

Coronary CT angiography (CCTA) allows for the direct quantification and characterization of atherosclerotic plaque for potential risk evaluation beyond the assessment of stenosis [11–13]. Furthermore, CCTA enables serial non-invasive monitoring of plaque progression [14–16] and therefore may help to identify patients at risk for future adverse cardiovascular events [17, 18].

The aim of the present study was to monitor the progression of the atherosclerotic coronary plaque burden by serial cardiac CT and evaluate its relationship with cardiac events in asymptomatic diabetic patients.

## Methods

### Study population

This study was approved by the Institutional Review Board of Chinese PLA general hospital with a waiver of informed consent due to the retrospective nature of this investigation. The authors had full control of the data and the information submitted for publication. We consecutively included asymptomatic patients who were referred to our hospital for evaluation and underwent serial CCTA between October 2011 and December 2015 as part of their clinical work-up for the evaluation of CAD.

Among all CCTA scans performed within the dedicated time period, 225 patients matched the following inclusion criteria: (1) a prior CCTA study performed at our institution during the study period that had identified no obstructive (i.e., ≥50% coronary stenosis) CAD; (2) no known history of obstructive CAD or an infectious/inflammatory cardiovascular condition (e.g., aortitis, Kawasaki disease); (3) diagnosis of DM including (a) men ≥50 years or women ≥55 years with ≥3 years history of DM (b) men ≥40 years or women ≥45 years with ≥6 years history of DM. Patients were classified into two groups based on CACS to reflect their cardiovascular risk: group A, CACS> 10 (high risk); group B, CACS≤10 (low risk) [19]. Exclusion criteria were symptomatic cerebral vascular disease or symptomatic peripheral vascular disease, known history of obstructive CAD with ≥50% coronary stenosis and/or prior coronary revascularization. Patient baseline characteristics including cardiac risk factors and clinical parameters were obtained from medical records.

### CCTA acquisition

All CCTA examinations were performed using a 2nd generation dual-source CT (DSCT) system (Somatom Definition Flash, Siemens Healthineers, Forchheim, Germany). For coronary artery calcium scoring, a non-contrast enhanced calcium scan was performed with the following parameters: collimation of 32 × 1.2 mm; 120kVp tube voltage; tube current of 75 mA; 3-mm slice thickness with 1.5 mm increment. Scan parameters for the subsequent contrast enhanced CCTA were as follows: collimation of 2 mm × 64 mm × 0.6 mm, z-axis flying focus technique and a gantry rotation of 280 ms. Patients with a body mass index (BMI) ≥26 kg/m^2^ were examined with a tube voltage of 120kVp, whereas patients with a BMI < 26 kg/m^2^ were examined with a tube voltage of 100kVp. Each tube provided a maximum of 430mAs/rotation. Weighted filtered back projection image reconstruction was performed in the cardiac phase with the least motion and the following parameters: section thickness of 0.75 mm, reconstruction increment of 0.5 mm and a smooth convolution kernel (B26f).

### Analysis of CCTA data and quantitative plaque assessment

CCTA data were transferred to a post-processing workstation (syngo.via VA30, Siemens Healthineers) for further analysis. Transverse sections and automatically generated curved multi planar reformations were available for assessment. CCTA data were analyzed in consensus by two experienced observers according to CAD reporting and data system (CAD-RADS) [20]. Each segment was assessed for interpretability. Segments were defined as uninterpretable in the presence of severe motion artefacts or low contrast opacification. Additionally, segments with a diameter < 2 mm were excluded from further analysis [7].

**Fig. 1:**
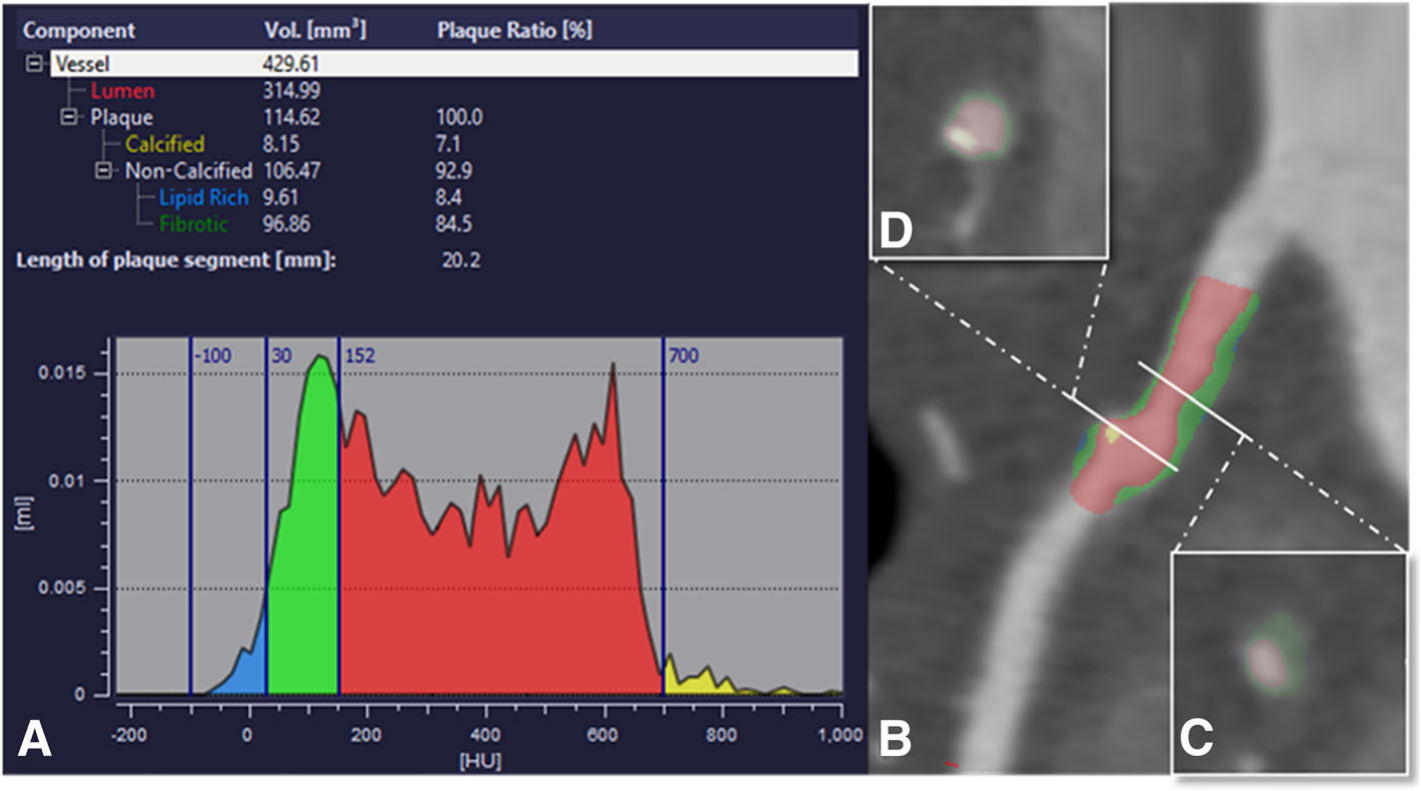
Coronary plaque analysis from computed tomography coronary angiography. To obtain quantitative and qualitative patient-specific plaque markers, the plaque burden is analyzed according to tissue attenuation based on Hounsfield Units (**a**). Example of the longitudinal tracking by an automatically generated curved multiplanar image data reformation along the vessel centerline (**b**) and corresponding transverse luminal sections perpendicular to the centerline (**c** and **d**) provided by the software

A dedicated semi-automatic software prototype (Coronary Plaque Analysis 2.0.3 syngo.via FRONTIER, Siemens) was used for the quantitative plaque analysis. This analytical software uses automated segmentation based on attenuation values in Hounsfield Units (HU) specific to the target anatomy within user-defined proximal and distal boundaries to compute quantitative atherosclerotic lesion descriptors. All automated contouring of the external vessel wall and internal lumen was reviewed on the basis of 0.5 mm sections. Manual contouring of the centerline, lumen, and vessel wall boundary was performed in case the automated segmentation provided by the software deviated from the correct vessel boundaries. Plaque volume was calculated by subtracting the lumen volume from the total vessel volume **(**Fig. 1**)**. Plaque volumes were then normalized by coronary artery length to determine the plaque volume index (PVI, in mm^2^). Plaque subtypes have previously been defined as “soft”, “lipid-rich” plaque (-100HU - 30HU), “fibrous” plaque (>30HU - 350HU), and “calcified” plaque (>350HU - 1300HU) and expressed as a percentage of overall plaque volume to generate the soft-, fibrous-, and calcified plaque fraction (in %).

“Vulnerable” plaques were defined as exhibiting at least 2 of the following 4 imaging biomarkers: 1) Positive remodeling above 10% calculated by the ratio of lesion diameter to a proximal reference diameter in a normal vessel segment. 2) Low attenuation, defined by having a mean attenuation < 150 HU in the plaque. 3) Spotty calcifications, defined as calcified nodules < 3 mm in size and not occupying more than 90 degrees of the coronary arc. 4) “Napkin-ring” sign, defined as a cross section with non-calcified plaque with a ring-like higher attenuation pattern surrounding a central area of lower attenuation in contact with the coronary lumen.

### Follow-up assessment

Follow-up data were prospectively gathered via phone-call between December 2017 and February 2018 by an independent investigator blinded to initial CCTA results. Medical records from both the department of cardiology and the referring outpatient diabetic clinic were analyzed. Three endpoints were recorded: 1. all-cause mortality, 2. non-fatal myocardial infarction, 3. revascularization. Non-fatal myocardial infarction was defined based on the criteria of typical chest pain, elevated cardiac enzyme levels, and changes on electrocardiogram that are typically associated with infarction. Revascularization was defined as receiving percutaneous coronary intervention or coronary artery bypass grafting after CCTA acquisition. A composite endpoint was constructed from all three endpoints for the analysis.

### Statistical analysis

Statistical analysis was performed using SPSS (SPSS 19.0, IBM, Chicago, USA). Continuous variables were expressed as mean ± SD, and discrete variables as absolute numbers and percentages. Student’s t-tests was used to evaluate differences in continuous variables between the two groups. A chi-squared test or Fisher’s exact test was used to test differences concerning categorical data and the Wilcoxon rank-sum test was used for non-normally distributed variables. MACE and their components were compared using multivariable logistic regression models to estimate odds ratios (OR) with 95% confidence intervals (CIs) and were plotted on Kaplan-Meier curves. A multivariable logistic regression model was created to predict cardiac events at the time of repeat CCTA based on age, sex, and all variables that showed a *p* value < 0.1 in the univariable analysis. The final model only included variables with a *p* value < 0.05 in the initial multivariable model. The selective inclusion of variables helped build a parsimonious model with a small number of variables and meaningful predictability for straightforward clinical implementation. Model fit of the final model was assessed by a Hosmer-Lemeshow-test and Pearson goodness-of-fit test, and the C-statistic was calculated as the discriminatory power of the model.

## Results

A total of 225 patients who underwent CCTA were included. Eighteen patients were excluded due to poor image quality, resulting in a final study population of 197 patients. All patients had DM with mean disease duration of 5.0 ± 9.7 years.47/197 patients (23.9%) used insulin, and a history of diabetic complications was present in 11 (5.6%). Eighty-five patients (43.1%) had hyperlipidemia, and 113 (57.4%) had hypertension. Sixty patients (30.4%) had a smoking history. Statins were used by 89 patients (45.2%). The mean BMI was 25.7 ± 3.3 kg/m^2^. Based on the pretest probability using updated Diamond-forrester methods, there was no difference in the distribution of pretest likelihood of CAD for both groups. Based on the CAD-RADS reporting guideline, the two groups showed insignificant difference in severity of coronary stenosis. Further patient demographics and baseline characteristics are presented in Table 1.

**Table 1:** Baseline characteristics and clinical demographics of the study population

	All patients (*n* = 197)	Patients with CACS ≤10 (*n* = 156)	Patients with CACS > 10 (*n* = 41)
Clinical characteristics			
Age, years (±SD)	63 ± 11.4	62.3 ± 11.3	66.1 ± 11.1
Males	125 (63.4%)	96 (61.5%)	29 (70.7%)
BMI, kg/m^2^ (±SD)	25.7 ± 3.3	25.8 ± 3.4	25.1 ± 3.1
Hypercholesterolemia	85 (43%)	72 (46.1%)	13 (31.7%)
Hypertension	113 (57.3%)	92 (58.9%)	21 (51.2%)
Smoking	60 (30.4%)	48 (30.7%)	12 (29.3%)
Family history of CAD	35 (17.8%)	26 (16.7%)	9 (21.9%)
Baseline CAC Score	12.5 [0, 102.5]	6.5 [0, 9.8]	29.2 [11.3, 102.5]
Pretest likelihood of CAD			
Low	57	45	12
Moderate	111	89	22
High	29	22	7
Medical therapy			
Aspirin	110 (52.7%)	82 (52.5%)	22 (53.7%)
Statins	71 (36.0%)	53 (34.0%)	18 (43.9%)
Β-blocker	81 (41.1%)	57 (46.5%)	24 (58.5%)
Calcium channel blocker	59 (29.9%)	46 (29.4%)	13 (31.7%)
CAD-RADS reporting data			
CAD-RADS 0	36	30	7
CAD-RADS 1	51	41	13
CAD-RADS 2	100	77	19
CAD-RADS 3	10	8	2

Values are shown as numbers with percentages in parentheses or mean values with standard deviation

The median time interval between the first and second CCTA was 18.9 months (Q1-Q3, 16.4–32.5 months). The change of plaque characteristics in both patient groups between serial CCTA acquisitions is shown in Table 2. Patients with CACS≤10 showed a total mean plaque volume increase of 20.6 (±63.5) mm^3^ annually between CCTA scans, resulting in an increase of 2.2%. The CACS> 10 group demonstrated an average total plaque volume increase of 30.1 (±102.6) mm^3^ annually between acquisitions, corresponding to an increase of 3%. Comparing the increase of total plaque volume between each group showed a significantly larger increase in the CACS> 10 group (*p* = 0.002). There was no statistically significant difference regarding the percent change of atheroma volume between the two groups (*p* = 0.676), with calculated increases of 5.7% (±3.9) and 4.2% (±3.6) in CACS≤10 group and CACS> 10 group, respectively.

**Table 2:** Change in coronary plaque composition over serial CCTA

Patients (*n* = 197)	Patients with CACS ≤10			Patients with CACS > 10			*P* value
	Baseline	Repeat	Δ-Value	Baseline	Repeat	Δ-Value	
Total lumen vol.	125.5 (±113.7)	125.48 (±107.0)	2.95 (±67.8)	182.3 (±131.8)	194.0 (±141.8)	10.8 (±110.3)	0.060
Total plaque vol.	85.8 (±89.4)	104.4 (±106.5)	20.6 (±63.5)	102.1 (±83.4)	132.1 (±116.5)	30.1 (±102.6)	0.002
PAV, % (±SD)	39.3 (±16.4)	44.0 (±16.4)	5.7 (±13.9)	34.7 (±12.8)	38.9 (±12.7)	4.2 (±13.6)	0.676
Total dense calcium							
Mean (±SD)	6.7 (±18.9)	6.8 (±16.4)	0.3 (±13.6)	23.1 (±28.0)	28.8 (±35.9)	5.7 (±28.5)	0.002
Median [IQR]	0 [0, 5.2]	0 [0,8.2]	0 [0,2.5]	12.2 [6.5,28.0]	17.2 [7.6,31.1]	1.43 [−0.81,4.11]	0.387
PAV, % (±SD)	2.5 (±4.5)	3.0 (±5.2)	0.6 (±2.5)	8.0 (±7.5)	9.1 (±8.8)	1.1 (±4.4)	0.360
Total fibrotic vol.							
Mean (±SD)	63.5 (±61.9)	71.1 (±63.0)	9.2 (±40.6)	65.1 (±49.5)	77.8 (±62.9)	12.6 (±53.3)	0.029
PAV, % (±SD)	30.9 (±14.2)	31.7 (±13.0)	0.9 (±12.9)	22.9 (±10.5)	23.7 (±11.3)	0.7 (±10.7)	0.70
Total low attenuation vol.							
Mean (±SD)	15.9 (±25.9)	29.7 (±49.6)	14.3(±34.9)	14.6 (±20.9)	25.5 (±35.0)	10.9 (±34.1)	0.018
PAV, % (±SD)	6.7 (±7.9)	10.2 (±10.0)	3.6 (±7.9)	4.3 (±4.7)	6.2 (±6.6)	1.8 (±7.4)	0.850

Continuous variables are presented as mean (±SD) and median [IQR] for the dense calcium volume. Percent Atheroma Volume (PAV) is presented as percentages. Volumes (Vol.) are measured in mm^3^. Differences between delta (Δ) values were tested with a student’s t-test (p) in normal distributed variables and in non-normal distributed variables with the non-parametric Kruskal-Wallis test (p*)

The CACS> 10 group showed a mean increase in dense calcium volume of 5.7 (±28.5) mm^3^ (23%) over baseline, whereas the dense calcium mean volume increased by 0.3 (± 13.6) mm^3^ in the group with CACS≤10, corresponding to an increase of 4.1%. The larger increase in dense calcium volume in the CACS> 10 group was statistically significant when compared to the CACS≤10 group (*p* = 0.029). Between acquisitions, the average volume of plaque with low “soft”, “lipid-rich” attenuation values (i.e., -100HU-30HU), increased by 14.3 (±34.9) mm^3^ in the CACS≤10 group, corresponding to an increase of 3.6% (±7.9) by percent atheroma volume. In patients with CACS> 10, the mean low attenuation plaque volume increased by 10.9 (±34.1) mm^3^, corresponding to an increase of 1.8% (±7.4) by percent atheroma volume. Significant differences between the groups regarding the change of mean low attenuation plaque volumes were observed (*p* = 0.018); however, there was no significant difference regarding the percent change of atheroma volume (*p* > 0.05).

The comparison of both groups showed a significant difference in the change of average total intermediate attenuation (i.e., >30HU - 350HU) “fibrotic” volume (*p* = 0.018), but no significant difference in percent change of atheroma volume (*p* > 0.05).

### Analysis of features of plaque vulnerability

**Fig. 2:**
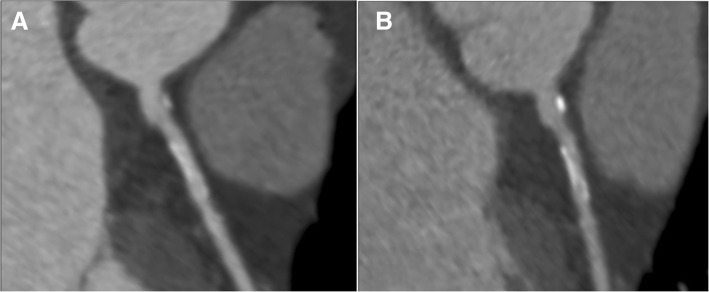
Example of coronary calcification progression at serial coronary computed tomography angiography. Automatically generated curved multiplanar reformation of image data of the left anterior descending coronary artery in an asymptomatic diabetic 60-year-old man performed in May 2011 (**a**) and in December 2013 (**b**). There is progression of dense calcification as well as overall plaque volume, while the stenosis of the coronary artery showed no obvious worsening

**Fig. 3:**
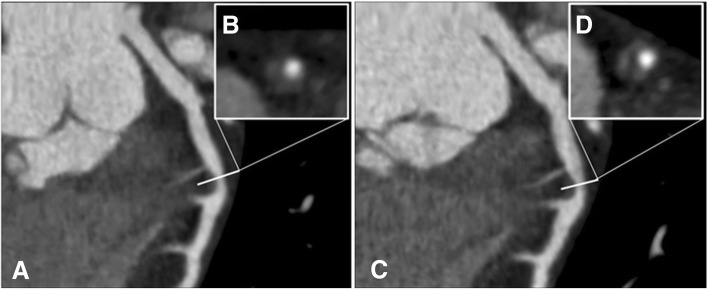
Example of non-calcified plaque progression at serial coronary computed tomography angiography. Automatically generated curved multiplanar reformation of image data of the left anterior descending coronary artery in an asymptomatic diabetic 46-year-old man performed in December 2010 (**a**) and in June 2013 (**c**). Displays of transverse sections perpendicular to the centerline show significant progression of the low-attenuation plaque volume from baseline (**b**) to follow-up (**d**) while the degree of coronary artery stenosis at this level worsens

Twenty plaques with at least 2 previously described biomarkers of plaque vulnerability were identified at baseline CCTA (12 in the CACS≤10 group and 8 in the CACS> 10 group), while 29 plaques with vulnerable morphology were identified at follow-up CCTA (18 in the CACS≤10 group and 11 in the CACS> 10 group). Case examples of the progression in the prevalence of plaque features of vulnerability are illustrated in Figs. 2 and 3. There was no statistically significant difference on the effects of coronary calcification on the rate of vulnerable plaque characteristics (*p* > 0.05).

### Follow-up analysis

**Fig. 4:**
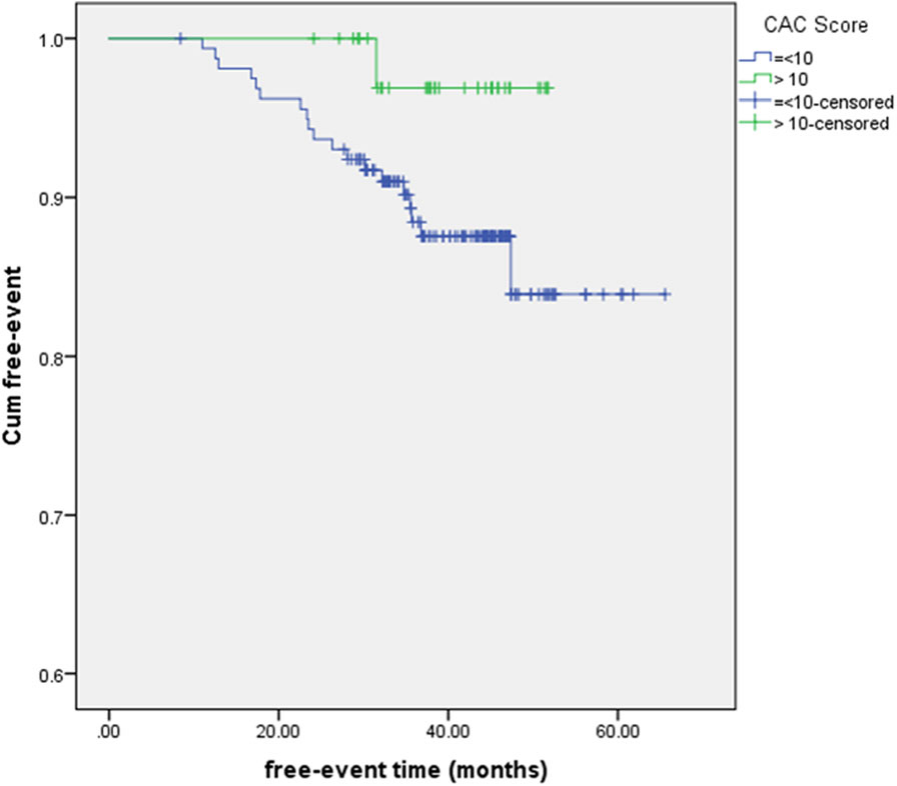
Event-free survival for the composite endpoint (all-cause mortality, non-fatal myocardial infarction, late revascularization) according to CAC-score

Follow-up was completed for all patients included in the study, with a median follow-up time of 41.8 months (Q1-Q3: 32.9–46.1 months). A total of 20 adverse cardiac events occurred (2 cardiac deaths, 15 non-fatal myocardial infarctions and 3 revascularizations). Within a follow-up period of 3 years, only 3 cardiovascular events occurred in subjects with CACS> 10, corresponding to an overall event-free survival rate of 98.8%. The majority of events (*n* = 17) occurred in subjects with CACS≤10. Event-free survival curves for patients in both groups are shown in Fig. 4. The cumulative event-free survival rates were 95% in patients with CACS> 10 and 81% in those with CACS≤10 (log-rank *p* = 0.025).

The final multivariable logistic predictive models are presented in Table 3. The final model included the presence of CACS> 10 at the time of repeat CCTA (adjusted odds ratio [OR], 0.701; 95% CI, 0.612–0.836), the increase in dense calcium volume (OR, 0.860 95% CI, 0.771–0.960), and the increase in “soft”, “lipid-rich” low attenuation plaque volume (OR, 1.013; 95% CI, 1.007–1.020) between the two CCTA scans. The model demonstrated good discriminatory power (C-statistic, 0.74) with a Hosmer-Lemeshow χ2 statistic of 1.94 (*p* value for lack of fit *p* = 0.75) and Pearson χ2 of 3.29 (*p* value for lack of fit *p* = 0.51). The bootstrapped C-statistic was 0.73 and the overfitting bias was 0.8%.

**Table 3:** Multivariate Logistic regression models for prediction of cardiac events

Variables	Initial Model			Final Model		
	OR	*p* value	95% CI	OR	*p* value	95% CI
Age	1.036	0.202	0.981–1.095	…	…	…
Male sex	1.825	0.343	0.527–6.322	…	…	…
Hypertension	2.786	0.130	0.738–10.511	…	…	…
Dyslipidemia	1.687	0.362	0.548–5.198	…	…	…
tobacco abuse	1.240	0.771	0.291–5.282	…	…	…
Family history CAD	3.353	0.063	0.936–12.013	…	…	…
CAC score > 10	0.554	0.017	0.505–0.589	0.701	0.034	0.612–0.836
Calcium Vol. change	0.804	0.004	0.693–0.933	0.860	0.007	0.771–0.960
Fibrotic Vol. Change	1.002	0.928	0.969–1.035	…	…	…
Lipid Vol. Change	1.016	0.009	1.004–1.027	1.013	< 0.001	1.007–1.020
Total Plaque Change	1.000	0.969	0.977–1.025	…	…	…

*CI* Confidence interval, *OR* Odds ratio, *Vol.* Volume

## Discussion

Our results showed that in our population of diabetics the change in calcium volume was inversely related to adverse cardiac outcomes. Additionally, the study results indicated that the low attenuation “lipid-rich”, soft plaque volume serves as an independent biomarker for predicting future cardiac events, progression of which was positively associated with adverse outcomes during follow-up. It has been argued that the plaque component exhibiting low-attenuation “lipid-rich” features as determined by CT to some extent represents the necrotic core of “vulnerable” atheromatous lesions [21]. Lastly, patients with a CACS> 10 included in the study demonstrated a higher cumulative event-free survival rate compared to patients with a CACS≤10.

The relationship between atherosclerotic plaque progression and cardiovascular risk in asymptomatic diabetic patients is insufficiently studied. Baseline CCTA demonstrated a moderate prevalence of coronary plaque burden (65%) in the study population, while patients with obstructive CAD at baseline were excluded from further analysis. During the follow-up CT scan, plaque quantification yielded different plaque progression patterns in the study subgroups. Specifically, using a median interval time of 18.9 months between CCTA acquisitions, total plaque volume increased by 20.6mm^3^ in the CACS≤10 group, compared to an increase of 30.1mm^3^ in the CACS> 10 group (*p* = 0.002). This statistically significant difference can be mostly attributed to the increase in total dense calcium volume (0.3mm^3^ vs. 5.7mm^3^, *p* = 0.029). Furthermore, low-attenuation plaque volume increased by 14.3mm^3^ in the CACS≤10 group, compared to an increase of 10.9 mm^3^ in the CACS> 10 group (*p* = 0.018). This significant increase in low-attenuation plaque volume in participants with CACS≤10 corresponded with a higher rate of adverse events in this patient group compared to subjects with CACS> 10. The more pronounced increase in low-attenuation plaque volume showed independent discriminatory power for the prediction of cardiac events on Cox regression analysis.

Several studies have evaluated the prognostic value of coronary artery calcium, stenosis severity, and cardiac events in diabetic patients. Results from these studies demonstrated an incremental increase in event rates corresponding with increasing calcium Agatston score and stenosis severity [9, 10, 22]. However, these studies failed to evaluate the association between calcification and plaque progression in the early disease stages of asymptomatic diabetics with non-obstructive CAD. Additionally, very few studies using serial CCTA for the evaluation of plaque progression in diabetic patients are available. Kim et al. demonstrated a 2.5-fold greater progression of total plaque volume during a mean scan interval of 3.8 years in diabetic vs. non-diabetic patient populations. More importantly, the progression of non-calcified plaque volume was significantly greater than that of calcified plaque volume [23]. The significant increase in total plaque volume and the increase of low-attenuation plaque material as a possible marker of necrotic core volume in the present study confirm these previous results.

A recent study focusing on the coronary plaque progression in diabetic patients showed that the baseline percentile of plaque volume and male sex were predictors of progression, but not the coronary calcification [23]. However, in present study the greater increase in low-attenuation plaque in the CACS≤10 group corresponded with a higher incidence of high-risk plaque features and eventual adverse cardiac events. The CACS> 10 group showed a significantly greater progression in total plaque volume, which was mainly due to the increase in total dense calcium. The discrepancy between higher calcium progression and lower cardiac event rates may be explained by plaque stabilization under statin therapy [24]. On a histo-pathologic level, asymptomatic patients with diabetes usually exhibit chronic endothelial damage and local factors that promote progressive atherosclerosis. These factors in conjunction with glycated protein oxidation could accelerate the calcification process, leading to spotty calcification associated with plaque vulnerability [25]. The present study illustrated a greater progression of spotty calcifications in patients with CACS≤10 than in patients with CACS> 10 while it appears conceivable that dense calcification may stabilize the plaque with a decrease in necrotic core burden and frequency of cardiac events.

Although CCTA can be a useful tool for risk stratification in diabetic patients with at least 5 years of asymptomatic CAD [26], it requires further investigation to determine whether the progression of plaque burden can be used for personalized treatment guidance and surveillance, especially in patients with high cardiovascular risk. Using serial CCTA acquisitions, our investigation demonstrated the feasibility of quantifying plaque progression and identified several imaging biomarkers that may have a positive or inverse association with future cardiac events in asymptomatic diabetics. Conceivably, repeat CCTA at an intermediate time-point after the initiation of optimized medical therapy may inform granular custom-tailoring of the treatment regimen based on the individual progression pattern of the coronary plaque burden. Although current Appropriate Use Criteria for Cardiac Computed Tomography do not incorporate prior CCTA results [27], the value of serial CCTA may warrant more consideration, especially for asymptomatic diabetic patients at high risk who may benefit from personalized risk stratification and modification of optimized medical therapy [28].

Several limitations to the present study deserve consideration. This is a single-center retrospective investigation with a patient population representing a single ethnicity, with potential bias due to selection and test power. It is possible that the prevalence and disease course of CAD could be influenced by the patient population’s ethnicity or other geographical factors. Furthermore, we did not systematically correlate our findings on coronary CTA with an invasive reference standard for intracoronary plaque assessment. Although the plaque software prototype has been validated in several studies [13, 29], there might be variation among different automated plaque quantification software. Thus, the same software should be used for serial assessment. Likewise, plaque quantification might be affected by technical influences, e.g. by the attenuation level of vascular contrast enhancement [30]. Moreover, the present study lacked of medication details in treating diabetes. New advanced treatment like incretin (GLP-1) therapy may play an important role in preventing plaque progression based on its pluripotent cardiovascular protective effects [31]. Also, the potential mechanism, for example the effect of genes’ polymorphism in secondary affecting the prognosis in the study population [32], needs to be further investigated. Therefore, these results may be applicable to very specific population, and prospective studies on larger study cohorts will be necessary to validate our findings.

## Conclusions

This investigation demonstrates that quantification of plaque progression derived from non-invasive serial CCTA portends incremental prognostic value, with low-attenuation “lipid-rich” atheromatous plaque volume showing the highest predictive value. Meanwhile, an increase of dense coronary artery calcification is inversely correlated with the progression of low-attenuation plaque burden, arguably reflecting the role of the pathophysiology of calcification on plaque stabilization during coronary atherosclerosis in diabetes. Granular serial evaluation of atheromatous plaque progression patterns may benefit asymptomatic diabetic patients via personalized risk re-stratification and subsequent adjustment of optimized medical therapy.

## Abbreviations

BMI: Body mass index
CACS: Coronary artery calcium score
CAD: Coronary artery disease
CCTA: Coronary CT angiography
CI: Confidence interval
DM: Diabetes mellitus
DSCT: Dual-source CT
MACE: Major adverse cardiac event
OR: Odds ratio
PVI: Plaque volume index
SD: Standard deviation
